# Immune effector cell-associated hematotoxicity following CAR-T cell therapies: insights from a large national database

**DOI:** 10.1093/jjco/hyag103

**Published:** 2026-07-01

**Authors:** Connor Frey

**Affiliations:** Department of Medicine, University of British Columbia, 2194 Health Sciences Mall, Vancouver, BC, V6T 1Z3, Canada

**Keywords:** CAR-T, immune effector cell-associated hematotoxicity, ICAHT, axicabtagene ciloleucel, Bayesian analysis

## Abstract

Immune effector cell-associated hematotoxicity is a recognized but incompletely characterized complication of chimeric antigen receptor T-cell (CAR-T) therapy, defined by prolonged, severe, or recurrent cytopenias following CAR-T infusion, not explained by conditioning chemotherapy or disease progression alone. Despite formal consensus grading criteria from the European Hematology Association and European Bone Marrow Transplantation group, the post-marketing pharmacovigilance profile of ICAHT across all approved CAR-T agents has not been systematically described. The FDA Adverse Event Reporting System (FAERS) was queried through March 2026 using OpenVigil 2.1 for all seven currently FDA-approved CAR-T products. Disproportionality was assessed using the Reporting Odds Ratio (ROR) with 95% confidence intervals, Proportional Reporting Ratio with chi-squared statistic, and Bayesian Reporting Rate Ratio. An adverse drug reaction classification was applied when all three signal detection thresholds were simultaneously met. ICAHT cases were identified in five of seven agents. Axicabtagene ciloleucel had the highest case count (*n* = 18) and most precisely estimated ROR (1365.54; 95% CI: 778.83–2394.24). All five agents with ICAHT reports met pre-specified signal detection criteria across all three analytical methods, with lower ROR confidence bounds substantially exceeding 1.00 in every case. No ICAHT reports were identified for obecabtagene autoleucel or tisagenlecleucel, with the former having minimal post-marketing data and the latter potentially reflecting genuine mechanistic differences related to its 4-1BB co-stimulatory domain. These findings demonstrate that ICAHT generates exceptionally strong and concordant pharmacovigilance signals across multiple CAR-T products, supporting heightened post-infusion hematologic monitoring with a low threshold for investigating unexplained cytopenias.

## Teaser

FAERS disproportionality analysis identified exceptionally strong, concordant ICAHT signals across five of seven FDA-approved CAR-T products, supporting heightened post-infusion hematologic surveillance and a low threshold for investigating unexplained prolonged cytopenias.

## Introduction

Chimeric antigen receptor T cell (CAR-T) therapy has transformed the treatment of relapsed or refractory hematologic malignancies, with seven products currently Food and Drug Administration (FDA)-approved across numerous indications [1]. The antitumor efficacy of these agents is driven by the *ex vivo* engineering of autologous T-cells to express a receptor targeting tumor-associated antigens, enabling potent redirected cytotoxicity upon reinfusion [2].

The same potency that underlies their therapeutic benefit confers a spectrum of toxicities unique to the CAR-T platform. Cytokine release syndrome and immune effector cell-associated neurotoxicity syndrome are the most extensively characterized, but recognition has grown that hematologic toxicity extending beyond the expected lymphodepletion conditioning regimen constitutes a distinct and clinically important complication [3]. Immune effector cell-associated hematotoxicity (ICAHT) is a recently formalized syndrome defined by prolonged, severe, or recurrent cytopenias occurring after CAR-T infusion and not fully explained by disease progression or conditioning chemotherapy alone [4].

ICAHT is clinically relevant because it is associated with serious infectious complications, transfusion dependence, prolonged hospitalization, and in severe cases, treatment-related mortality. The European Bone Marrow Transplantation and European Hematology Association have issued consensus grading criteria for ICAHT, distinguishing early-onset (within 30 days) from late-onset forms and incorporating neutrophil and platelet nadirs into severity grading [4]. Despite this formal recognition, the post-marketing pharmacovigilance profile of ICAHT across all approved CAR-T agents has not been systematically characterized in the FDA Adverse Event Reporting System (FAERS), the largest spontaneous adverse drug reaction (ADR) reporting database in the world. A disproportionality analysis of FAERS was conducted through March 2026 to quantify ICAHT reports for all approved CAR-T products.

## Methods

### Data source

Adverse event reports were extracted from FAERS using OpenVigil 2.1, a publicly accessible pharmacovigilance platform, from database inception through March 2026; the methods have been previously described [5]. FAERS aggregates spontaneous ADR reports from healthcare professionals, manufacturers, and consumers worldwide and is maintained by the FDA. The drugs of interest were all currently FDA-approved CAR-T cell therapy products: axicabtagene ciloleucel, brexucabtagene autoleucel, ciltacabtagene autoleucel, idecabtagene vicleucel, lisocabtagene maraleucel, obecabtagene autoleucel, and tisagenlecleucel. Each agent was queried by generic name. The primary outcome was the MedDRA Preferred Term “immune effector cell-associated hematotoxicity”.

### Statistical analysis

Disproportionality was assessed using the Reporting Odds Ratio (ROR) with 95% confidence intervals (CIs), calculated for each agent individually. The ROR compares the odds of a specific adverse event being reported for a given drug relative to all other drugs in the database. A signal was considered statistically significant when the lower bound of the 95% CI exceeded 1.00. The Proportional Reporting Ratio (PRR) and its associated χ^2^ statistic were additionally calculated; a PRR ≥2 with χ^2^ ≥ 4 is a widely used threshold for signal detection. The Bayesian Reporting Rate Ratio (RRR) was calculated as a complementary measure of disproportionality, with values >1 indicating disproportionate reporting. An ADR classification was applied when all three criteria were met: PRR ≥2, χ^2^ ≥ 4, and ROR 95% CI lower bound >1.

## Results

ICAHT was identified in five of seven approved CAR-T agents (Table 1). The highest absolute case count was observed with axicabtagene ciloleucel (*n* = 18 of 4909 total reports; 0.37%), followed by ciltacabtagene autoleucel (*n* = 7 of 3096; 0.23%), brexucabtagene autoleucel (*n* = 3 of 760; 0.39%), and idecabtagene vicleucel and lisocabtagene maraleucel each with one report (0.27% and 0.31%, respectively). No cases of ICAHT were identified for obecabtagene autoleucel or tisagenlecleucel, both of which have substantially smaller total report volumes in FAERS relative to the other agents.

**Table 1:** Immune effector cell-associated hematotoxicity reports across FDA-approved CAR-T agents.

**CAR-T agent**	**Antigen**	**Reports**	**PRR**	**χ** ^**2**^	**ADR**	**ROR**	**95% CI**	**RRR**
Axicabtagene ciloleucel	CD19	18	1360.54	15 683.58	Yes	1365.54	778.83, 2394.24	923.54
Brexucabtagene autoleucel	CD19	3	1050.45	2066.43	Yes	1054.61	328.83, 3382.24	994.23
Ciltacabtagene autoleucel	BCMA	7	650.69	3424.96	Yes	652.16	295.17, 1440.91	569.47
Idecabtagene vicleucel	BCMA	1	698.76	170.58	No	700.66	96.70, 5076.69	686.30
Lisocabtagene maraleucel	CD19	1	784.23	191.57	No	786.64	108.53, 5701.47	770.25
Obecabtagene autoleucel	CD19	–	–	–	–	–	–	–
Tisagenlecleucel	CD19	–	–	–	–	–	–	–

PRR = proportional reporting ratio (signal threshold: ≥2 with χ^2^ ≥ 4); ROR = reporting odds ratio; 95% CI = 95% confidence interval; RRR = Bayesian reporting rate ratio; – = not available.

All five agents with reported ICAHT cases demonstrated profoundly elevated PRR values (Table 1). Axicabtagene ciloleucel generated the highest PRR (1360.54) with a χ^2^ statistic of 15 683.58, reflecting both the strength of the signal and the relatively larger denominator of total reports. Brexucabtagene autoleucel produced a PRR of 1050.45 (χ^2^ 2066.43), and ciltacabtagene autoleucel a PRR of 650.69 (χ^2^ 3424.96). Idecabtagene vicleucel and lisocabtagene maraleucel, each with a single ICAHT report, nevertheless generated PRR values of 698.76 and 784.23, respectively, with χ^2^ statistics of 170.58 and 191.57. The ROR for axicabtagene ciloleucel was 1365.54 (95% CI: 778.83–2394.24), representing the most precisely estimated effect due to the larger case count. Brexucabtagene autoleucel produced an ROR of 1054.61 (95% CI: 328.83–3382.24) and ciltacabtagene autoleucel an ROR of 652.16 (95% CI: 295.17–1440.91). For the single-case agents, CIs were necessarily wide: idecabtagene vicleucel (ROR 700.66, 95% CI: 96.70–5076.69) and lisocabtagene maraleucel (ROR 786.64, 95% CI: 108.53–5701.47). Critically, in all five cases, the lower bound of the 95% CI substantially exceeded 1.00, confirming statistical significance. Bayesian RRR values corroborated these signals, exceeding 500 in all five agents and approaching 1000 for brexucabtagene autoleucel (RRR 994.23) and axicabtagene ciloleucel (RRR 923.54).

## Discussion

This pharmacovigilance analysis of FAERS demonstrates that ICAHT generates exceptionally strong disproportionality signals across CAR-T agents in the two largest product classes. The magnitude of the signals observed, with ROR point estimates exceeding 600 in all five agents and lower confidence bounds far exceeding 1.00, is consistent with a true pharmacological association rather than reporting artifact, and the concordance across both frequentist and Bayesian approaches strengthens this interpretation.

The biological plausibility of ICAHT is well established. Unlike the cytopenias arising directly from lymphodepletion conditioning with fludarabine and cyclophosphamide, ICAHT is driven by a distinct mechanism involving CAR-T cell-mediated disruption of hematopoiesis. Proposed mechanisms include direct cytotoxic effects of activated CAR-T cells on hematopoietic progenitors expressing low-level target antigen, macrophage activation and inflammatory cytokine-mediated suppression of the bone marrow niche, and impairment of hematopoietic stem cell engraftment [4,6]. In BCMA-targeting therapies, additional potential mechanisms include BCMA expression on plasma cells and plasmablasts that are integral to the bone marrow microenvironment, potentially rendering the hematopoietic niche more susceptible to collateral damage during BCMA-directed CAR-T activity [4,7,8].

The observation that axicabtagene ciloleucel carries the highest absolute report count and most precisely estimated ROR likely reflects both its longer post-marketing experience and its broader approved indications across multiple lymphoma subtypes, which collectively generate a larger FAERS denominator and more opportunity for ICAHT event capture. The higher PRR observed for brexucabtagene autoleucel relative to ciltacabtagene autoleucel, despite fewer total reports, may reflect indication-specific differences.

The absence of ICAHT reports for obecabtagene autoleucel and tisagenlecleucel warrants careful interpretation. Tisagenlecleucel, approved for pediatric and young adult acute lymphoblastic leukemia and relapsed/refractory follicular lymphoma, has the second-largest total report volume in this dataset, yet no ICAHT cases were identified. This may reflect genuine differences in the hematotoxicity profile of this agent: tisagenlecleucel uses a 4-1BB co-stimulatory domain rather than the CD28 domain used by axicabtagene ciloleucel and brexucabtagene autoleucel, a distinction associated with different T-cell persistence kinetics and potentially different patterns of cytokine secretion and bone marrow engagement [9,10]. Obecabtagene autoleucel, the most recently approved agent in the dataset, has by far the smallest total report volume (*n* = 11), making the absence of ICAHT reports uninformative with respect to true signal absence; as post-marketing experience accumulates, this warrants re-evaluation.

This analysis carries the inherent limitations of spontaneous pharmacovigilance databases, including underreporting, the inability to establish causality at the individual-case level, confounding by indication, variable hematologic monitoring protocols across treatment centers, and potential MedDRA coding inconsistencies as the ICAHT preferred term is relatively newly adopted in clinical practice. The small case counts for idecabtagene vicleucel and lisocabtagene maraleucel limit the precision of the ROR estimates for those agents, and the absence of true denominators precludes calculation of absolute ICAHT incidence rates. Despite these limitations, disproportionality analysis is a validated hypothesis-generating tool in post-marketing surveillance, and the convergence of signal strength across all three analytical methods, across five distinct CAR-T products argues strongly for the validity of the signal identified here.

These findings have direct clinical implications. Hematologic monitoring protocols following CAR-T infusion should account for the possibility of late-onset ICAHT, and treating physicians should maintain a high index of suspicion for prolonged cytopenias that are not explained by conditioning or disease status alone. Early hematology consultation and consideration of growth factor support, eltrombopag, or hematopoietic stem cell top-up may be warranted in severe or refractory cases.

## Data Availability

The data are freely available through the FDA Adverse Events Reporting System Database and OpenVigil 2.1.
